# Pandemic influenza A virus codon usage revisited: biases, adaptation and implications for vaccine strain development

**DOI:** 10.1186/1743-422X-9-263

**Published:** 2012-11-08

**Authors:** Natalia Goñi, Andrés Iriarte, Victoria Comas, Martín Soñora, Pilar Moreno, Gonzalo Moratorio, Héctor Musto, Juan Cristina

**Affiliations:** 1 Laboratorio de Virología Molecular, Centro de Investigaciones Nucleares, Facultad de Ciencias, Universidad de la República, Iguá 4225, Montevideo, 11400, Uruguay; 2 Laboratorio de Organización y Evolución del Genoma, Instituto de Biología, Facultad de Ciencias, Universidad de la República, Iguá 4225, Montevideo, 11400, Uruguay; 3 Laboratorio de Evolución, Instituto de Biología, Facultad de Ciencias, Universidad de la República, Iguá 4225, Montevideo, 11400, Uruguay; 4 Unidad de Proteínas Recombinantes, Institut Pasteur de Montevideo, Mataojo 2020, Montevideo, 11400, Uruguay; 5 Unidad de Biofísica de Proteínas, Institut Pasteur de Montevideo, Mataojo 2020, Montevideo, 11400, Uruguay

**Keywords:** Influenza A virus, Codon usage, Evolution

## Abstract

**Background:**

Influenza A virus (IAV) is a member of the family *Orthomyxoviridae* and contains eight segments of a single-stranded RNA genome with negative polarity. The first influenza pandemic of this century was declared in April of 2009, with the emergence of a novel H1N1 IAV strain (H1N1pdm) in Mexico and USA. Understanding the extent and causes of biases in codon usage is essential to the understanding of viral evolution. A comprehensive study to investigate the effect of selection pressure imposed by the human host on the codon usage of an emerging, pandemic IAV strain and the trends in viral codon usage involved over the pandemic time period is much needed.

**Results:**

We performed a comprehensive codon usage analysis of 310 IAV strains from the pandemic of 2009. Highly biased codon usage for Ala, Arg, Pro, Thr and Ser were found. Codon usage is strongly influenced by underlying biases in base composition. When correspondence analysis (COA) on relative synonymous codon usage (RSCU) is applied, the distribution of IAV ORFs in the plane defined by the first two major dimensional factors showed that different strains are located at different places, suggesting that IAV codon usage also reflects an evolutionary process.

**Conclusions:**

A general association between codon usage bias, base composition and poor adaptation of the virus to the respective host tRNA pool, suggests that mutational pressure is the main force shaping H1N1 pdm IAV codon usage. A dynamic process is observed in the variation of codon usage of the strains enrolled in these studies. These results suggest a balance of mutational bias and natural selection, which allow the virus to explore and re-adapt its codon usage to different environments. Recoding of IAV taking into account codon bias, base composition and adaptation to host tRNA may provide important clues to develop new and appropriate vaccines.

## Background

Influenza A virus (IAV) is a member of the family *Orthomyxoviridae* and contains eight segments of a single-stranded RNA genome with negative polarity [1]. IAV is one of the most important infectious diseases in humans [2]. Unlike most pathogens where exposure leads to lasting immunity in the host, IAV presents a moving antigenic target [3], evading specific immunity triggered by previous infections. This process, called antigenic drift, is the result of the selective fixation of mutations in the gene encoding the hemagglutinin (HA) protein and to a lesser extent in the neuraminidase (NA) protein [4]. Variants that best escape the host immune response are thought to have a significant reproductive advantage [5]. Another process, called reassortment, is also considered a major force in the evolution of IAV [4]. It occurs when the virus acquires an HA and/or NA of a different IAV subtype (via reassortation) of one or more gene segments. This process has been in the basis of the devastating influenza pandemics that occurred several times in the last century [6].

The first influenza pandemic of this century was declared in April of 2009, with the emergence of a novel H1N1 IAV strain (H1N1pdm) in Mexico and USA [7,8]. By November of 2009, the virus was detected in about 207 countries, infecting more than 620,000 individuals worldwide and accounting for more than 7,800 deaths [7]. This strain was a multiple reassortant with genes derived from viruses that originally circulated in the swine, avian and human populations [9].

It has been observed that IAV is subjected to host immune selection pressure and undergoes rapid evolution, especially when the virus crosses the host species barrier [10]. The replication cycle of IAV depends on host machinery and the virus utilizes host cellular components for its protein synthesis. Therefore, the interplay of codon usage of virus and host could affect viral replication. For these reasons, a detailed understanding of IAV evolution and host adaptation is crucial.

Due to the degeneracy of the genetic code, most amino acids are coded by more than one codon. Synonymous triplets are not used randomly. In several organisms, natural selection and mutational input seem to bias codon use toward a certain subset of codons [11]. Two major models have been proposed to explain codon usage: the translational selection and the mutational models [12]. Codon usage bias related to translation efficiency (at two different levels: speed and accuracy) seems to be linked to local cognate isoacceptors tRNAs abundances, which in turn determine the major codon preferences [13]. On the other hand, discrepancies on codon usage could be due to genome compositional constraints and mutational biases [14]. Nevertheless, these two models cannot be considered as mutually exclusive.

Although previous studies have been performed on the general codon usage of IAV [2,12,15,16], a deep and comprehensive study to investigate the effect of selection pressure imposed by the human host on the codon usage of an emerging, pandemic IAV strain and the trends in viral codon usage involved over the pandemic time period is much needed.

In order to gain insight into these matters, we performed a comprehensive codon usage analysis of 310 H1N1pdm IAV strains, isolated from April to September of 2009, for which the complete genome sequences are available.

## Results

In order to study the extent of codon usage bias in H1N1pdm IAV strains in relation to seasonal H1N1 and H3N2 as well as human and swine host cells, the relative synonymous codon usage (RSCU) [14] values for each codon were calculated for the 310 H1N1pdm strains enrolled in these studies and compared with seasonal IAV strains and host organisms. The results of these studies are shown in Table 1.

**Table 1:** **Codon usage in 2009 H1N1 pdm Influenza A Virus, displayed as RSCU**^***a ***^**values**

**AA**	**Cod**	**HC**	**Swine**	**H1N1pdm**	**H1N1**^***b***^	**H3N2**	**AA**	**Cod**	**HC**	**Swine**	**H1N1pdm**	**H1N1**	**H3N2**
Phe	UUU	0.92	0.79	0.85	0.98	0.96	Ser	UCU	1.14	0.99	1.08	1.12	0.91
	UUC	1.08	1.21	1.15	1.02	1.04		UCC	1.32	1.50	0.74	0.87	0.97
Leu	UUA	0.48	0.32	0.62	0.91	0.62		**UCA**	**0**.**90**	**0**.**73**	**1**.**57**	**1**.**62**	**1**.**34**
	UUG	0.78	0.67	1.00	1.27	1.30		*UCG*	*0*.*30*	*0*.*39*	*0*.*31*	*0*.*14*	*0*.*21*
	CUU	0.78	0.65	1.16	0.97	1.24	Pro	CCU	1.16	1.05	1.00	1.04	1.29
	CUC	1.20	1.35	0.95	0.59	0.78		CCC	1.28	1.46	0.80	0.72	0.84
	**CUA**	**0**.**42**	**0**.**33**	**1**.**20**	**1**.**00**	**0**.**96**		**CCA**	**1**.**12**	**0**.**94**	**1**.**70**	**1**.**74**	**1**.**29**
	CUG	2.40	2.68	1.07	1.27	1.11		*CCG*	*0*.*44*	*0*.*56*	*0*.*50*	*0*.*49*	*0*.*58*
Ile	AUU	1.08	0.91	1.07	1.07	1.03	Thr	ACU	1.00	0.83	1.01	1.11	1.28
	AUC	1.41	1.67	0.77	0.78	0.89		ACC	1.44	1.68	0.79	0.96	0.72
	**AUA**	**0**.**51**	**0**.**42**	**1**.**16**	**1**.**16**	**1**.**08**		**ACA**	**1**.**12**	**0**.**92**	**1**.**88**	**1**.**74**	**1**.**67**
Met	AUG	1.00	1.00	1.00	1.00	1.00		*ACG*	*0*.*44*	*0*.*57*	*0*.*32*	*0*.*19*	*0*.*34*
Val	GUU	0.72	0.57	0.83	0.97	1.06	Ala	GCU	1.08	0.96	0.98	1.13	1.06
	GUC	0.96	1.07	0.77	0.74	0.69		GCC	1.60	1.80	0.87	0.87	0.93
	**GUA**	**0**.**48**	**0**.**34**	**1**.**12**	**1**.**07**	**1**.**02**		**GCA**	**0**.**92**	**0**.**74**	**1**.**87**	**1**.**74**	**1**.**73**
	GUG	1.84	2.03	1.28	1.22	1.23		*GCG*	*0*.*44*	*0*.*50*	*0*.*27*	*0*.*26*	*0*.*28*
Tyr	UAU	0.88	0.73	1.04	1.09	1.13	Cys	UGU	0.92	0.79	0.88	1.09	0.79
	UAC	1.12	1.27	0.96	0.91	0.87		UGC	1.08	1.21	1.12	0.91	1.21
TER	UAA	**	**	**	**	**	TER	UGA	**	**	**	**	**
	UAG	**	**	**	**	**	Trp	UGG	1.00	1.00	1.00	1.00	1.00
His	**CAU**	**0**.**84**	**0**.**70**	**1**.**23**	**1**.**05**	**1**.**21**	Arg	*CGU*	*0*.*48*	*0*.*44*	*0*.*11*	*0*.*24*	*0*.*10*
	CAC	1.16	1.30	0.77	0.95	0.79		*CGC*	*1*.*08*	*1*.*31*	*0*.*33*	*0*.*18*	*0*.*24*
Gln	**CAA**	**0**.**54**	**0**.**44**	**1**.**05**	**1**.**33**	**1**.**36**		*CGA*	*0*.*66*	*0*.*60*	*0*.*63*	*0*.*41*	*0*.*43*
	CAG	1.46	1.56	0.95	0.67	0.64		*CGG*	*1*.*20*	*1*.*29*	*0*.*43*	*0*.*28*	*0*.*57*
Asn	AAU	0.94	0.79	1.15	1.20	1.15	Ser	AGU	0.90	0.77	1.14	1.15	0.95
	GAC	1.08	1.21	0.95	0.80	0.85		AGC	1.44	1.62	1.16	1.11	1.38
Lys	AAA	0.86	0.76	1.10	1.27	1.39	Arg	**AGA**	**1**.**26**	**1**.**12**	**2**.**89**	**3**.**08**	**2**.**84**
	AAG	1.14	1.24	0.90	0.73	0.61		**AGG**	**1**.**26**	**1**.**23**	**1**.**61**	**1**.**81**	**1**.**83**
Asp	GAU	0.92	0.80	1.05	1.13	1.08	Gly	GGU	0.64	0.57	0.57	0.60	0.69
	GAC	1.08	1.20	0.95	0.87	0.92		GGC	1.36	1.46	0.62	0.55	0.62
Glu	**GAA**	**0**.**84**	**0**.**72**	**1**.**20**	**1**.**15**	**1**.**14**		**GGA**	**1**.**00**	**0**.**91**	**1**.**73**	**1**.**84**	**1**.**65**
	GAG	1.16	1.28	0.80	0.85	0.86		GGG	1.00	1.05	1.08	1.01	1.04

^*a*^*RSCU*, relative synonymous codon usage; *AA*, amino acid; *Cod*, codons; *HC*, human cells; *H1N1pdm*, 2009 H1N1 pdm Influenza A virus; H1N1 and H3N2, seasonal H1N1 and H3N2 Influenza A virus, respectively. Highly increased codons with respect to host cells (∆ ≥ 0.30) are shown in bold. Codons containing de dinucleotide CG are shown in italics. ^*b*^ RSCU codon usage of seasonal H1N1 and H3N2 according to Wong et al. (2010) [12].

All codons containing the dinucleotide CpG were underrepresented in all IAV viruses. Important differences were found between human and swine hosts and IAV strains. Particularly, high biased frequencies (∆ RSCU ≥ 0.30) were found for Leu, Ile, Val, Ser, Pro, Thr, Ala, His, Gln, Glu, Arg and Gly. Interestingly, the huge majority of preferred codons in the viruses are A-ended. In the case of Arg, there is a strong bias towards an increase in AGA and AGG, while the CGN codons are depleted (see Table 1).

To observe if H1N1pdm IAV strain sequences display similar codon usage biases, the effective number of codons (ENC) [17] values were calculated for the 310 strains enrolled in this study (mean of 52.51 ± 0.05). ENC varies from 20 to 61, where the larger the extent of codon bias in a gene, the smaller the ENC value. Thus, a value of 52.5 strongly suggests that the overall codon usage among these strains is only slightly biased.

**Figure 1:**
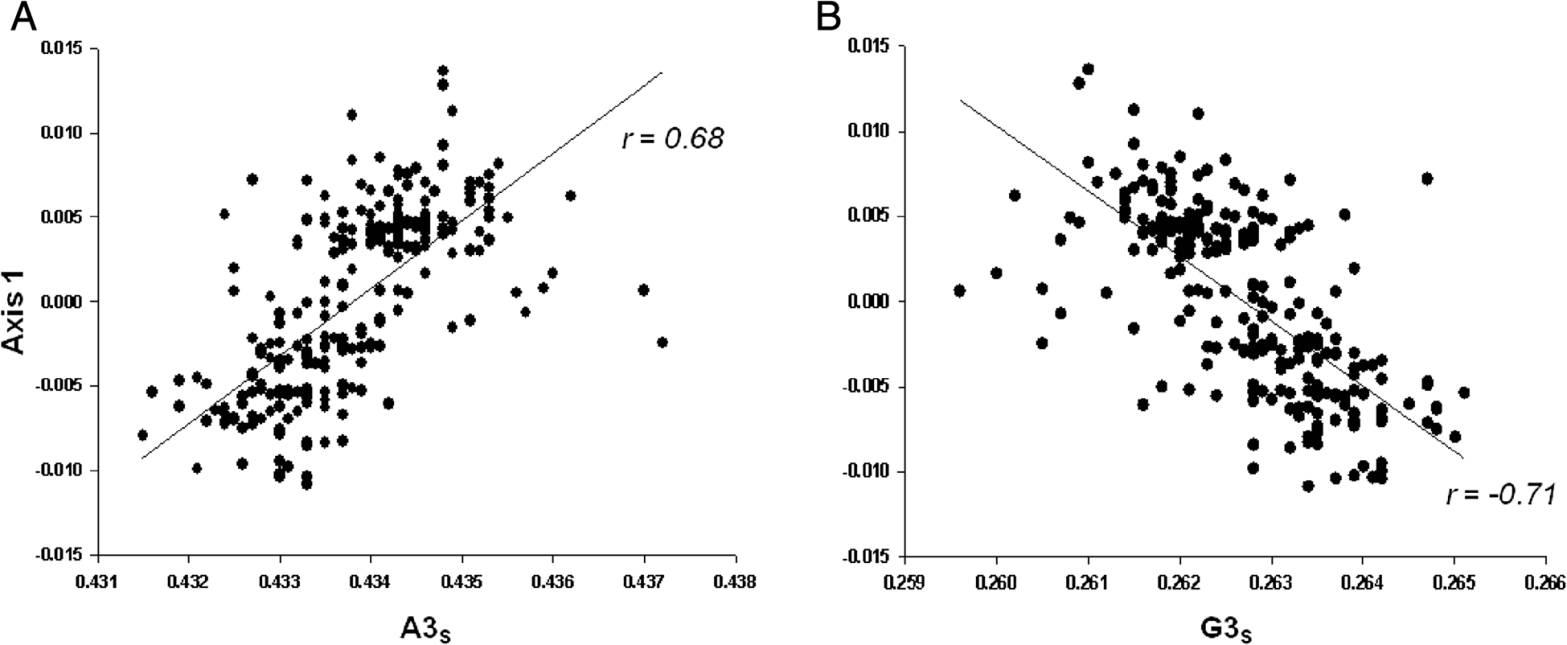
**Association of purines at third codon positions with dimensional factor 1 generated by COA.** In (**A**) and (**B**), the regression plots of the frequency of A3s and G3s *versus* the respective position of each strain in the first dimensional factor generated by the correspondence analysis on RSCU (COA-RSCU) are shown.

Since codon usage by its very nature is multivariate, it is necessary to analyze the data using multivariate statistical techniques, like correspondence analysis (COA) [18]. The correlation between the position on the first dimensional factor generated by this analysis on RSCU (20.7% of the total variability) for each strain and the respective G + C content at synonymous variable third position (GC_3_s) values was significant (r = −0.47, p < 0.0001). Interestingly, this dimensional factor also significantly correlated with A content at synonymous variable third position (A_3_s, r = 0.68, p < 0.0001) and G content at the same position (G_3_s, r = −0.71, p < 0.0001) (Figure 1). This means that the major factor shaping codon usage among these strains is an opposite trend between purines at third codon positions. Furthermore, this result is mainly due to the frequencies of the codons CGA (Arg) on one side of the distribution and GCG (Ala) and CGG (Arg) at the other side (see Additional file 1: Table S1). In other words, the differential usage of three low frequent codons (RSCU ≤ 0.63) is among the major factor shaping codon usage among these strains.

It has been suggested that dinucleotide biases can affect codon bias [19]. To study the possible effect of dinucleotide composition on codon usage of the H1N1pdm IAV strains, the relative abundances of the 16 dinucleotides in the ORFs of the 310 strains enrolled in these studies were established. The results of these analyses are shown in Table 2.

**Table 2:** **Summary of correlation analysis between the dimensional factors** (**DF**) **in COA and sixteen dinucleotides frequencies in H1N1 pdm IAV ORFs**

		**UU**	**UC**	**UA**	**UG**	**CU**	**CC**	**CA**	**CG**
Mean ± SD^*a*^		0.893 ± 0.0054	0.814 ± 0.0050	0.736 ± 0.0009	1.215 ± 0.0009	0.797 ± 0.0056	0.672 ± 0.0033	1.326 ± 0.0042	0.319 ± 0.0020
DF 1^*b*^	*r*	0.43277	0.30726	0.50328	0.49116	0.16033	0.40283	0.44451	0.47789
	*P*	<0.0001	<0.0001	<0.0001	<0.0001	0.0048	<0.0001	<0.0001	<0.0001
		**AU**	**AC**	**AA**	**AG**	**GU**	**GC**	**GA**	**GG**
Mean ± SD^*a*^		1.281 ± 0.0046	0.926 ± 0.0039	1.804 ± 0.0071	1.327 ± 0.0037	0.682 ± 0.0076	0.703 ± 0.0009	1.472 ± 0.0012	1.040 ± 0.0018
DF 1^*b*^	*r*	0.44790	0.36540	0.61328	0.40489	0.08304	0.49579	0.48484	0.45555
	*P*	<0.0001	<0.0001	<0.0001	<0.0001	0.11880	<0.0001	<0.0001	<0.0001

^*a*^ Mean values of 310 H1N1 pdm IAV strains’ relative dinucleotide ratios ± standard deviation. ^*b*^ Correlation analysis between the first dimensional factor in COA and the sixteen dinucleotides frequencies in H1N1 pdm IAV ORF’s is shown.

As it can be seen in the table, the occurrences of dinucleotides are not randomly distributed and no dinucleotides were present at the expected frequencies (Table 2). The relative abundance of CpG showed a strong deviation from the “normal range” (mean ± S.D. = 0.319 ± 0.0020) and were markedly underrepresented. Interestingly, when the second dimensional factor (11.1% of the total variability) was analyzed, we found that the position of each strain significantly correlated (r = 0.64, p < 0.0001) with the respective usage of the dinucleotide CpG. Besides, although the global usage of this dinucleotide is very low, we found that the correlation is due to the differential usage of CGU (Arg) and CCG (Pro) codons, since these triplets display the most extreme values on the second dimensional factor (see Additional file 1: Table S1). Importantly, we also found that the third and the fourth dimensional factors of COA (8.7% and 5.5% of the total variability, respectively), are again mainly linked to the low usage of codons containing the dinucleotide CpG, mainly at the positions 2 and 3. Moreover, among the 16 dinucleotides, 15 are highly correlated with the first dimensional factor value in COA (Table 2). These observations indicate that the composition of dinucleotides also plays a crucial role in the variation found in synonymous codon usage among H1N1pdm IAV ORFs.

**Figure 2:**
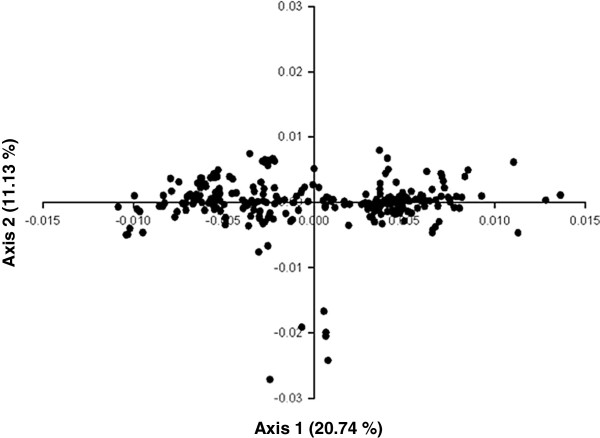
**Position of the 310 H1N1 pdm IAV ORF’s in the plane defined by the first two major axes generated by COA.** The percentage of inertia of the first and second axes of COA is indicated for both axes between parentheses. The input values for COA were the RSCU values of each strain.

To study the possibility of codon usage variation in the H1N1pdm IAV genomes enrolled in this study, the distribution of the 310 strains in the plane defined by the first two axes of COA was established. The results of these studies are shown in Figure 2.

Interestingly, the distribution of the H1N1pdm IAV strains in the plane defined by the first two major axes showed that the principal dimensional factor splits the strains at least three major groups: two of them discriminated by the first dimensional factor, while the third is revealed by the extreme low values on the second dimensional factor (Figure 2).

**Figure 3:**
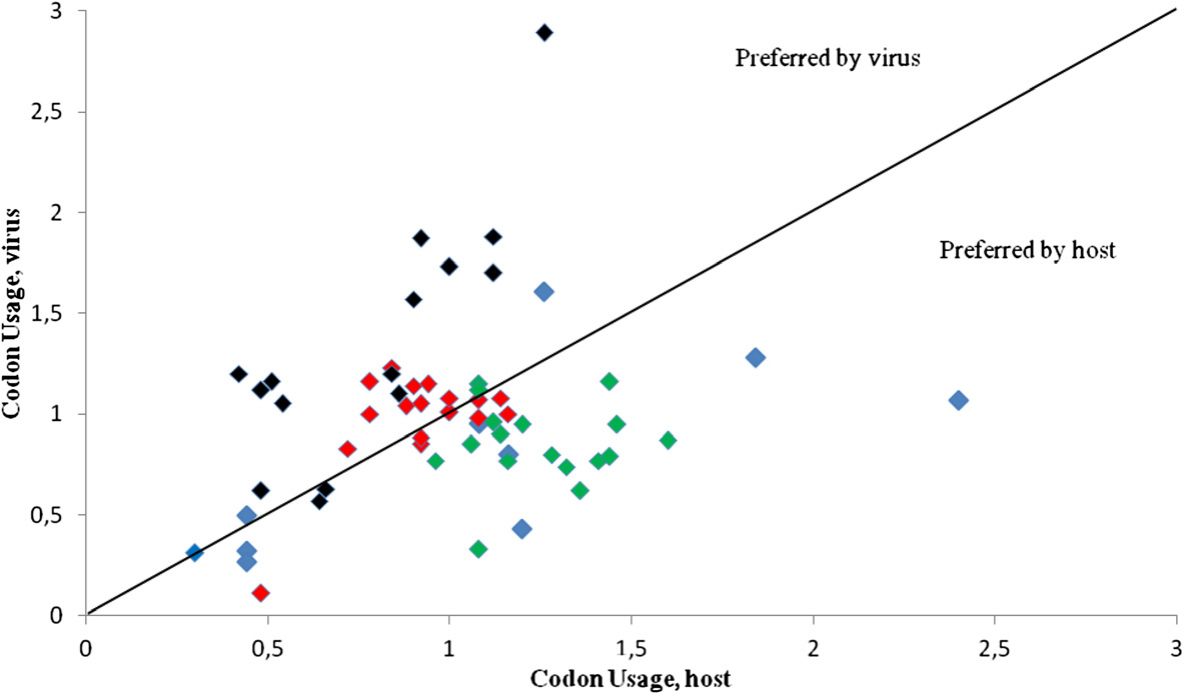
**Codon usage of H1N1 pdm IAV plotted against the codon usage of human cells.** Colors reflect the nucleotide that occupies the first anticodon position (wobble position) of the corresponding codon. A, C, G and T are indicated by red, blue, green and black diamonds, respectively.

As the translation process represents a key step in the viral infection cycle, it is important to explore the strategies employed by the virus to harness the translation machinery of the cell host. Since variation at the third codon position makes possible the wobble interaction between that base and the first one of the anticodon [20], we wanted to gain further insight into the adaptation of H1N1pdm IAV strains to the respective host tRNA pool context. For this reason, the codon usage of virus (H1N1pdm IAV) was plotted against the codon usage of host (human cells) and the nucleotide that occupy the first anticodon position (wobble position) of the corresponding codon was identified. The results of these studies are shown in Figure 3.

As it can be seen in the figure, codon usage of virus and host is uncorrelated. The viral preference toward AT rich genomes and the T-headed anticodons is clear (Figure 3). This is also in agreement with the consequence of a differential usage of A3_s_ and G3_s_ (see also Figure 1). Comparison of these findings with the compilation of tRNAs species in the human genome [21] reveals that the virus highly preferred T-headed anticodons are not particularly adapted to the host transfer tRNA pool (Table 3). Therefore, there is no obvious correlation between the number of human host isoacceptor tRNAs and codon usage of the IAV enrolled in these studies.

**Table 3:** Frequency of tRNA genes in human cells for highly biased codons in H1N1 pdm IAV*

**AA**	**Cod**	**Anticodon isotypes** (**tRNA count by anticodon**)	**Total tRNA anticodon count**
Ala	**GCA**	**UGC**(**9**), AGC(29), GGC(0), CGC(5)	43
Arg	**AGA** &**AGG**	**UCU**(**6**), **CCU**(**5**), ACG(7), GCG(0), CCG(4), UCG(6)	28
Gln	**CAA**	**UUG**(**11**), CUG(21)	32
Glu	**GAA**	**UUC**(**13**), CUC(13)	26
Gly	**GGA**	**UCC**(**9**), GCC(15), CCC(7), ACC(0)	31
His	**CAU**	**AUG**(**0**), GUG(11)	11
Ile	**AUA**	**UAU**(**5**), AAU(14), GAU(8)	27
Leu	**CUA**	**UAG**(**3**), AAG(12), CAG(10), CAA(7), UAA(7), GAG(0)	39
Pro	**CCA**	**UGG**(**7**), AGG(10), GGG(0), CGG(4)	21
Ser	**UCA**	**UGA**(**5**), AGA(11), GGA(0), CGA(4), ACU(0),GCU(8)	28
Thr	**ACA**	**UGU**(**6**), AGU(10), GGU(0), CGU(6)	22
Val	**GUA**	**UAC**(**5**), CAC(16), AAC(11), GAC(0)	32

* Highly biased codons in H1N1 pdm IAV (as defined in Table 1) and their respective anticodons are shown in bold. *AA*, amino acid; *Cod*, codons.

## Discussion

As IAV relies on the host cell’s machinery for its replication, codon usage bias could play a role in its adaptation to the host. The results of these studies revealed that codon usage in human IAV, including H1N1pdm, do not have the average codon usage pattern of their host’s genes (see Table 1), in agreement with previous reports [12,16].

Comparisons to previous results reported for other IAV such H5N1 (mean ENC = 50.91) [16,22]; or other RNA viruses like SARS (mean ENC = 48.99) [23]; foot-and-mouth disease virus (mean ENC = 51.42) [24]; classical swine fever virus (mean ENC = 51.7) [19], Duck Enteritis virus (mean ENC =52.17) [25], Encephalomyocarditis virus (mean ENC = 54.86) [26] or Theilovirus (mean ENC = 51.08) [26], revealed that the ENC values found in this study for H1N1pdm IAV strains (mean ENC value of 52.5) are roughly similar to these previous findings, indicating that the overall extent of codon usage in these viruses are only slightly biased.

We have found a general link between codon usage bias and base composition, which is shown by the significant correlation of the position of each virus on the first dimensional factor of COA *vs*. the corresponding GC_3_s, together with the opposite trends in relation to purines at third codon position (Figure 1A and B). Taken together, our results indicate that the mutational bias is a very important trend in the evolution of H1N1pdm IAV genomes. However, this does not *per se* discards a role of other natural selection mechanisms acting in the IAV strains enrolled in these studies.

We have also found that CpG containing codons are sharply suppressed (see Table 1). This CpG deficiency was proposed to be related to the immunostimulatory properties of unmethylated CpG, which are recognized by the innate immune system of the host as a pathogen signature [24,27]. This is triggered by the intracellular Pattern Recognition Receptor (PRR) Tool-like 9 (TLR9), which activates several immune response pathways [28]. It seems reasonable to suggest that exists among vertebrates a TLR9-like mechanism acting at the RNA level [29]. Interestingly, previous studies have shown that IAV strains originated from an avian reservoir and infecting human hosts since 1918 has been selected under strong pressure to reduce the frequency of CpG in its genome [30]. Marked CpG deficiency has been observed in several other RNA viruses [24,31-35], including H1N1pdm IAV [12,30]. Then, escaping from the host antiviral response may act as another selective pressure contributing to codon usage in H1N1pdm IAV strains [36].

The distribution of the 310 H1N1 pdm IAV ORF’s in the plane defined by the first two axes of COA shows the presence of at least three clusters of strains (Figure 2). Since species with a close genetic relationship always present a similar codon usage pattern [37] (see also Table 1), the results of these studies suggests that a dynamic process occurred in the H1N1pdm strains enrolled in these studies. This is reflected in the variation of codon usage observed among them (see Figure 2). These results suggest a balance of mutational bias and natural selection to shape codon usage in these strains, which allow the virus to explore and re-adapt its codon usage to different environments in a short period of time.

From the classical point of view, the preferred codons are recognized by the most abundant isoacceptors tRNAs, which implies the action of natural selection [38]. The results shown in Table 3 strongly suggest that this is not the case for H1N1pdm IAV strains. In other words, codon usage of these viruses does not seem to be adapted to the tRNA pool of the human cells but probably reflects the influence of mutational biases. Interestingly, this has been observed for some other RNA viruses, like HIV [39].

Understanding the mechanisms used by IAV to properly express its genes could suggest a novel point of intervention and drug targets. Reduced translation efficiency, particularly of structural genes that are needed for the formation of new particles, could affect viral success [40].

The results of this work suggest that synthetic attenuated virus engineering (SAVE) could play a role in creating new vaccines for IAV. By deoptimization of codon usage (replacing wild-type codons with codons and codon combinations whose sequences impair replication and/or expression), it might be possible to attenuate a virus [41]. Moreover, as the codon changes do not alter the protein sequence, the antigenicity should not differ from the wild-type virus. Besides, codon changes tend to have individually small fitness effects, so many nucleotide changes will be required to restore wild-type fitness, itself requiring 100 s or more generations [42-45]. This “death by a thousand cuts” strategy may provide an alternative method of attenuation [46]. Interestingly, it has been show that replacement of natural codons with synonymous triplets with increased frequencies of CpG gives rise to inactivation of Poliovirus infectivity [47]. Very recent studies revealed that this strategy can be applied to IAV [48].

Owing to known genome sequences, modern strategies of DNA synthesis have made it possible to recreate in principle all known viruses independent of natural templates [48]. Recoding of IAV to develop new vaccine candidates taking into account codon bias, base composition and adaptation to host tRNA by gene synthesis may provide important clues to elucidate virulence factors, identify targets for future drug intervention, and to develop new and appropriate vaccines [49].

## Methods

### Sequences and dataset

Sequences from H1N1pdm IAV strains, isolated from April to December of 2009, were obtained from The Influenza Virus Resource at the National Center for Biotechnological Information [50]. The data set comprised the complete genome sequences (eight segments) of 310 strains. For each strain the ORFs were concatenated (PB2 + PB1 + PA + HA + NP + NA + MP + NS) and aligned using the MUSCLE program [51]. The alignment is available upon request.

### Codon usage analysis

Codon usage, base dinucleotide composition, G + C at synonymous variable third position codons (GC_3_s), the relative synonymous codon usage (RSCU) [14] and the effective number of codons (ENC) [17] were calculated using the program CodonW (written by John Peden and available at http://sourceforge.net/projects/codonw/) as implemented in the Mobile server ( http://mobyle.pasteur.fr). Codon usage data of influenza viral hosts, human (*Homo sapiens*) and domestic swine (*Sus scrofa*) were obtained from the codon usage database (available at: http://www.kazusa.or.jp/codon)[52]. The frequencies of tRNAs in human cells were retrieved from the GtRNAdb database [21].

### Correspondence analysis(COA)

COA is an ordination technique that identifies the major trends in the variation of the data and distributes genes along continuous axes in accordance with these trends. COA creates a series of orthogonal axes to identify trends that explain the data variation, with each subsequent dimensional factor explaining a decreasing amount of the variation [18]. Each ORF is represented as a 59-dimensional and each dimension is related to the RSCU value of each triplet (excluding AUG, UGG and stop codons). This was done using the CodonW program.

### Statistical analysis

Correlation analysis was carried out using Spearman’s rank correlation analysis method [53].

## Competing interests

The authors declare that they do not have competing interests.

## Authors’ contributions

JC conceived of the study, and participated in its design and coordination. NG and AI have made substantial contributions to the design of the study, acquisition of data and analysis. VC, MS, GM, GC, have been involved in revising the manuscript critically for important intellectual content. JC wrote the paper. HM helped to draft the manuscript and made substantial and fundamental contributions to the interpretation and discussion of the results found in this work. All authors read and approved the final manuscript.

## Supplementary Material

Additional file 1 **Table S1.** Each codon included in the correspondence analysis is represented by a row. Factor 1 and 2 columns contain the coordinate of the codon on the respective generated axis. click here for file
